# The Associations among Cyberbullying Victimization and Chinese and American Adolescents’ Mental Health Issues: The Protective Role of Perceived Parental and Friend Support

**DOI:** 10.3390/ijerph21081069

**Published:** 2024-08-15

**Authors:** Michelle F. Wright

**Affiliations:** Department of Psychology, Indiana State University, Terre Haute, IN 47809, USA; michelle.wright@indstate.edu

**Keywords:** cyberbullying, victimization, social support, friends, parents, depression, subjective health complaints, self-harm, mental health

## Abstract

Researchers have focused on identifying factors that may mitigate the negative consequences associated with cyberbullying victimization. A significant factor that has received considerable attention is perceived social support from parents and friends and its potential to reduce the risk of cyberbullying victimization and the associated negative mental health issues. However, the buffering effects of perceived social support from parents and friends on the longitudinal relationships among cyberbullying victimization, depression, subjective health complaints, and self-harm have been less explored, particularly in cross-cultural contexts. To address this gap, the present study examined the role of perceived social support from parents and friends in buffering against depression, subjective health complaints, and self-harm, measured one year later, associated with cyberbullying victimization among 463 Chinese (49% female) and 445 American (52% female) eighth graders (ages 13–15). They completed self-report questionnaires on cyberbullying victimization, perceived social support from parents and friends, and mental health (i.e., depression, subjective health complaints, self-harm). One year later, they completed the same mental health questionnaires. The findings revealed no differences in reports of perceived support from parents, but greater reports of social support from friends for American adolescents when compared to Chinese adolescents. High levels of perceived social support from parents were associated with a stronger negative relationship between cyberbullying victimization, depression, subjective health complaints, and self-harm for both Chinese and American adolescents, with these effects being more pronounced for Chinese adolescents, while opposite patterns were found for American adolescents and perceived social support from friends. These results are discussed in the context of cultural values and how these values shape the role of adults in adolescents’ lives.

## 1. Introduction

Information and communication technologies (ICTs) are deeply embedded in modern society, bringing numerous conveniences to our lives. However, they also expose users to various risks [1]. One significant risk associated with ICT use is cyberbullying victimization, which is often linked to depression, subjective health complaints, and self-harm [2]. Given the positive relationship between cyberbullying victimization, depression, and other negative outcomes, researchers are keen to identify factors that might mitigate the negative impacts of cyberbullying. One such factor is perceived social support from parents and friends. This study aims to examine cultural differences in perceived social support from parents and friends, as well as the buffering effect of such support, in the associations among cyberbullying victimization, depression, subjective health complaints, and self-harm among Chinese and American adolescents. 

### 1.1. Cyberbullying Victimization and Negative Outcomes

Cyberbullying victimization involves being victimized by repetitive and hostile behaviors through digital technologies, including information and communication technologies (ICTs) [3,4,5,6,7]. Similar to traditional face-to-face bullying, cyberbullying involves an imbalance of power between the bully and the victim and features repetitive actions [3,7,8]. However, the use of ICTs adds complexity, as it allows harmful content to be disseminated widely and rapidly. For instance, a perpetrator might post an embarrassing video of a victim online, which can then be shared extensively, intentionally or unintentionally, by countless viewers. Cyberbullying victimization behaviors often include rumor spreading, harassment, social exclusion, humiliation, physical threats, gossip, and verbal insults. Additionally, they involve physical forms such as hacking, making anonymous phone calls, forwarding explicit videos, identity theft or impersonation, and harassment via instant messaging, social media, and text messages [9,10]. Although adolescents can be involved in cyberbullying as perpetrators and bystanders, this study focuses exclusively on victimization. 

Victims can experience cyberbullying at almost any time, often involving multiple bystanders. Cyberbullying victimization is associated with various negative adjustment difficulties such as depression, anxiety, loneliness, and poor academic performance [11,12,13,14]. Additionally, multiple studies have demonstrated positive relationships between cyberbullying victimization and both suicidal ideation and non-suicidal self-harm [11,15]. Research has also explored the link between cyberbullying and subjective health concerns, such as stomachaches and headaches. In one of the pioneering studies on this topic, Kowalski and Limber [6] found that adolescent victims of cyberbullying reported more sleeping issues, headaches, poor appetite, and skin problems compared to their uninvolved peers. These findings have been replicated among Swedish and Finnish adolescents [16,17]. Sourander and colleagues [17] further discovered that cybervictims and cyberbullies–victims experienced more somatic complaints than uninvolved adolescents. Even after controlling for traditional face-to-face bullying victimization and computer use, Vieno et al. [18] and Laftman et al. [19] found that cyberbullying victimization was linked to somatic health issues for both adolescent boys and girls. Considering the negative outcomes associated with cyberbullying victimization, researchers have directed their attention to factors that can buffer against such negative consequences. Perceived social support is one such factor considered in the research literature. 

### 1.2. Perceived Social Support

Social support refers to the understanding that someone is cared for, valued, and part of a network concerned with their well-being [20,21,22]. This support, which can be physical, social, or psychological, plays a crucial role in enhancing feelings of security and self-worth during challenging times. Parents are often the primary source of social support, but as children grow into adolescents and young adults, they increasingly rely on friends for this support [23]. Extensive research highlights the positive impact of social support in mitigating face-to-face bullying. Victims of bullying often have poorer-quality friendships, which directly affects their access to help and protection [22,24,25,26]. These victims are frequently isolated from social interactions, resulting in poor social relationships that heighten their vulnerability to bullying. Studies have found a positive association between low perceived social support from family and peers and face-to-face victimization [9]. After experiencing a negative event, it is crucial for adolescents to have access to physical, social, and psychological support. This type of support helps them feel cared for and reassured that someone is there for them during challenging times and difficult situations. Adolescents frequently identify their parents as a significant source of social support [27].

Similarly, perceived social support reduces the risk of involvement in cyberbullying [27,28,29,30,31,32]. For instance, research by Williams and Guerra [33] found that adolescents who believed they had caring friends were less likely to be involved in cyberbullying. Having a social companion or someone who provides comfort also decreases the risk of cyberbullying. Even when friends are not supportive, family support can protect adolescents from cyberbullying. Notably, perceived parental support has been shown to have a stronger protective effect against cyberbullying than support from friends [34]. There are few studies on the buffering effects of perceived social support from parents and friends on the relationship between cyberbullying involvement and depression and other mental health issues, such as subjective health complaints and self-harm [21]. In face-to-face bullying research, social support has been shown to buffer against the negative effects of depression for victims. For example, Holt and Espelage [35] found that victims with moderate parental support had lower levels of depression compared to those with low perceived parental support. 

It is unknown how perceived social support from parents and friends might mitigate the negative impact of cyberbullying victimization on Chinese and American adolescents’ depression, subjective health complaints, and self-harm. Perceived social support from parents and friends plays a crucial role in the lives of adolescents, impacting their emotional and psychological well-being [34]. The nature of this support, however, can vary significantly between cultural contexts, such as those of American and Chinese adolescents. In American culture, which is often characterized by individualistic values, parental support emphasizes independence and self-reliance [36]. American adolescents may perceive parental support as encouragement towards independence and personal achievement [37]. Parents in the United States typically offer both emotional support and practical assistance. Emotional support includes expressing love, understanding, and empathy, while practical support involves providing resources, advice, and guidance [38]. In contrast, Chinese culture is rooted in collectivist values, where family cohesion and interdependence are prioritized. Filial piety, a core Confucian value, emphasizes respect and obedience towards parents and elders [39,40]. This cultural norm means that Chinese adolescents may perceive parental support as more authoritative and directive. Chinese parents often focus on academic achievement and moral development, and the support provided is frequently related to educational guidance and instilling discipline [41].

As American adolescents grow older, they tend to spend more time with their peers, who become a primary source of social support. Peer support in the United States is characterized by shared activities, emotional sharing, and mutual support in navigating social and academic challenges [42]. Friends often provide a platform for emotional expression and sharing personal experiences, contributing significantly to emotional well-being and social development [43]. While peer support is also important in China, it often revolves around academic collaboration and collective success. Friends may work together on school projects and study, providing mutual academic support [44]. Although less emphasized compared to American adolescents, emotional sharing among friends still exists but may be influenced by the cultural context of maintaining harmony and face [45]. Understanding these cultural differences in perceived social support can help educators, parents, and policymakers tailor interventions to better support adolescents’ well-being in diverse cultural contexts. Recognizing the unique ways in which support is manifested in American and Chinese cultures allows for more effective and culturally sensitive approaches to fostering adolescent development.

### 1.3. The Present Study

Based on studies exploring the distinct roles of parents and friends in the lives of Chinese and American adolescents, it is anticipated that these groups may influence adolescents’ perception of social support. This present study aims to investigate the disparities in perceived support from parents and friends between Chinese and American adolescents. Eighth-grade students are at a critical developmental stage, transitioning from early to middle adolescence. This period is marked by significant physical, emotional, and social changes, making adolescents particularly vulnerable to mental health issues. At this age, peer relationships become increasingly important, and the influence of social dynamics, including cyberbullying, is heightened. Furthermore, eighth graders are typically at the end of their middle school experience, a time when they are preparing for the transition to high school, which can add additional stressors. By focusing on this age group, this study can provide insights into the unique challenges faced by adolescents during this pivotal time, thereby enhancing the relevance and applicability of its findings.

It is hypothesized that Chinese adolescents will report higher levels of perceived social support from parents compared to their American counterparts, reflecting cultural values that emphasize respect for elders [39,40]; on the other hand, American adolescents will report greater perceived social support from friends than Chinese adolescents.

While existing research has examined how perceived social support from parents and friends affects cyberbullying victimization, little research has explored whether such support can mitigate the adverse effects of cyber victimization on adolescents’ mental health outcomes like depression, subjective health complaints, and self-harm. Depression is a pervasive and debilitating mental health issue that significantly impacts adolescents’ overall well-being, academic performance, and social interactions. Subjective health complaints, which encompass a range of physical and psychological symptoms, often serve as early indicators of stress and mental health problems, reflecting the broader impact of cyberbullying on adolescents’ daily lives. Self-harm is a critical and alarming behavior that highlights the severe consequences of cyberbullying, emphasizing the urgent need for effective interventions. By detailing the importance of these outcomes, this study’s findings underscore the necessity of addressing cyberbullying to safeguard adolescent mental health.

Nevertheless, evidence suggesting that parental and friend support reduces the risk of cyber victimization implies that parents and friends might also buffer or diminish the impact of cyberbullying victimization on adolescents’ psychosocial adjustment difficulties. Thus, it is hypothesized that higher levels of perceived social support from parents and friends will moderate these relationships, weakening the associations between cyberbullying victimization and depression as well as subjective health complaints and self-harm. Conversely, lower levels of perceived social support are expected to strengthen these associations. This hypothesis is expected to apply to both Chinese and American adolescents, with these associations being stronger for Chinese adolescents and perceived social support from parents and for American adolescents and perceived social support from friends. This study addresses the following research questions:

1. What are the differences in reported perceived social support from parents and friends between Chinese and American adolescents?
2. To what extent does perceived social support from parents and friends influence the associations among cyberbullying victimization, depression, subjective health complaints, and self-harm, and do these relationships vary between Chinese and American adolescents after controlling for face-to-face bullying victimization?

These questions aim to deepen the understanding of how parental mediation practices differ across cultures and their potential role in mitigating the negative impacts of cyberbullying on adolescent well-being. This study utilized a longitudinal design. A longitudinal study can reveal how perceived social support from parents and friends evolves over time between Chinese and American adolescents, providing insights into cultural differences and their impacts on adolescent development. Examining the long-term effects of perceived social support from parents and friends on the relationships between cyberbullying victimization, depression, subjective health complaints, and self-harm can help identify protective factors and cultural variations, offering targeted interventions for Chinese and American adolescents.

## 2. Materials and Methods

### 2.1. Participants

Participants in this study comprised 8th-grade middle school students from Beijing, China, and Chicago, Illinois, USA. The Beijing sample included 463 students (49% female, average age 14.01 years), while the Chicago sample included 445 students (52% female, average age 13.67 years). Participants attended schools situated in predominantly middle-class neighborhoods. Most Chinese participants identified as Han ethnicity. Among the American participants, 73% were White, 20% Latino/a, 5% Black/African American, 1% Asian, and 1% biracial. No income data were collected for this study. 

### 2.2. Procedures

This study was approved by the author’s university’s IRB and ethical standards were followed throughout the conduction of this study. The selection process involved randomly choosing ten public schools from a pool of over three hundred in both Chicago and Beijing. Initial contact with school principals was made via email, reaching out to five principals in each country, resulting in four Chinese and three American principals expressing interest. These schools were chosen to be representative of typical public schools in their respective countries. Face-to-face meetings were arranged with interested principals and teachers to discuss their rights as participants in this study, study objectives, and procedures. Following this, announcements were made in 7th-grade classrooms, accompanied by the distribution of parental permission slips detailing study participation requirements. In China, 501 permission slips were distributed, with 483 returned granting permission and 10 returned without permission. In the United States, 550 slips were distributed, resulting in 470 permissions granted and 25 declined. Three American participants were excluded due to absence on data collection days, with no absences among Chinese participants. Data collection occurred during the fall of the 7th grade (Time 1). Prior to the participants providing assent for this study, trained research assistants explained the confidentiality and privacy of the participants’ data and described their rights as participants in this study. Participants provided assent and completed demographic information (gender, age, ethnicity), along with measures of face-to-face and cyberbullying victimization, perceived social support from parents and friends, depression, subjective health complaints, and self-harm. Trained research assistants facilitated questionnaire administration in English for American participants and using back-translation techniques for Chinese participants.

One year later, during the fall of the 8th grade (Time 2), a reminder letter was sent to parents/guardians of previous participants, affirming their child’s ongoing participation. No letters were returned, and data collection proceeded with 463 Chinese and 445 American adolescents after excluding 20 Chinese and 25 American adolescents due to logistical reasons (e.g., relocation, absences). They completed questionnaires on perceived social support from parents and friends, depression, subjective health complaints, and self-harm.

### 2.3. Measures

#### 2.3.1. Face-to-Face Bullying Victimization

Adolescents in this study were surveyed to assess the frequency of face-to-face victimization experiences using a scale ranging from 1 (not at all) to 5 (all of the time) during the current school year. The measure consisted of twelve items, including examples such as “Someone gossiped about me” and “Someone called me insulting names” [46]. For Chinese adolescents, Cronbach’s alpha reliability coefficient for this measure was 0.90, indicating high internal consistency. Among American adolescents, Cronbach’s alpha was 0.94, demonstrating similarly strong reliability. This assessment of face-to-face bullying victimization was conducted exclusively at Time 1 of this study.

#### 2.3.2. Cyberbullying Victimization

Adolescents were asked to rate the frequency of cyberbullying victimization experiences on a scale from 1 (not at all) to 5 (all of the time) for incidents occurring online or through text messages within the current school year [14]. This measurement tool comprised nine items, such as “Someone sent me a nasty message online or through text messages” and “Someone called me insulting names online or through text messages”. The Cronbach’s alpha coefficient for cyberbullying victimization was α = 0.91 for both Chinese and American adolescents, indicating strong internal consistency. Cyberbullying victimization was assessed solely at Time 1 of this study.

#### 2.3.3. Perceived Social Support from Parents and Friends

Adolescents’ perceived social support from parents and friends was measured using two subscales. The subscale for perceived social support from friends consisted of 12 items, such as “My friends understand my feelings”. Similarly, the subscale for perceived social support from parents included items like “My parent or parents show they are proud of me” [26]. Responses for all items ranged from 1 (never) to 6 (always). Scores for each subscale were computed by averaging responses across relevant items. Higher scores indicated higher levels of perceived social support from parents and friends. The Cronbach’s alpha coefficients among Chinese adolescents were 0.85 at Time 1 and Time 2 for perceived social support from parents and 0.84 at Time 1 and 0.86 at Time 2 for perceived social support from friends. For American adolescents, the reliability was 0.86 for Time 1 and 0.85 for Time 2 for perceived social support from parents and 0.87 for Time 1 and Time 2 for perceived social support from friends.

#### 2.3.4. Depression

Depression was assessed using the Center for Epidemiological Studies Depression Scale [47], a measure comprising twenty items rated on a scale from 0 (rarely or none of the time) to 3 (most or all of the time). Sample items included “I was bothered by things that usually don’t bother me” and “I did not feel like eating, my appetite was poor”. The Cronbach’s alpha coefficients were 0.85 at Time 1 and Time 2 for Chinese adolescents and 0.88 at Time 1 and Time 2 for American adolescents, indicating good internal consistency. This questionnaire was administered at both Time 1 and Time 2 to assess changes in depression over the study period.

#### 2.3.5. Subjective Health Complaints

The Health and Behaviour in School-aged Children Symptom Checklist, developed by Haugland and Wold [48], was employed to evaluate the prevalent health symptoms experienced by the adolescents, regardless of medical diagnosis. Participants rated the frequency of eight health complaints over the preceding six months, including headache, stomachache, backache, feeling low, feeling irritable or bad-tempered, feeling nervous, difficulties in falling asleep, and dizziness, using a scale ranging from 1 (rarely or never) to 5 (about every day). Scores across these items were aggregated to derive a composite score reflecting overall subjective health complaints, where higher scores indicated greater levels of reported health issues. The checklist was administered at both Time 1 and Time 2 of this study. Cronbach’s alphas for this measure were 0.83 at Time 1 and Time 2 for Chinese adolescents and 0.81 at Time 1 and 0.83 at Time 2 for American adolescents, demonstrating good internal consistency across both assessment periods.

#### 2.3.6. Self-Harm

The Self-Harm Inventory, developed by Sansone et al. [49], was utilized to assess non-suicidal self-harm behaviors among adolescents. This instrument consisted of 22 yes/no items, including behaviors such as hitting oneself, purposefully cutting, and preventing wounds from healing. Responses to these items were aggregated to compute a total score ranging from 0 to 22, reflecting the extent of non-suicidal self-harm engagement. The inventory was administered at both Time 1 and Time 2 of this study. For American adolescents, Cronbach’s alphas were 0.83 at each assessment point. Among Chinese adolescents, the Cronbach’s alpha was 0.81 at Time 1 and 0.84 at Time 2, indicating good internal consistency across both groups and time points.

#### 2.3.7. Technology Use

Ten items were utilized to evaluate adolescents’ technology use, encompassing queries such as frequency of text messaging. Responses were rated on a scale from 1 (never) to 5 (all the time). These items were aggregated to generate a composite score reflecting overall technology use, where higher scores indicated more extensive engagement with technology. The internal consistency of the scale, measured by Cronbach’s alpha, ranged from 0.86 for Chinese adolescents and 0.88 for American adolescents, demonstrating good reliability.

### 2.4. Analytic Plan

To address this study’s research questions, structural equation modeling was employed using the robust maximum likelihood estimator and the full information maximum likelihood approach to handle missing data, which amounted to approximately 0.1% of the dataset, affecting two Chinese adolescents and two American adolescents. Missing data were analyzed; between two to four of the data points were missing for the endogenous variables. Little’s MCAR test was conducted for missing value analyses. Findings revealed that the data for the endogenous variables were not systematically missing (χ2 = 70.99, df = 81, *p* = 0.802), indicating that full information maximum likelihood estimation could be used for missing data in this study. Additional pathways were introduced from Time 1 cyberbullying victimization to Time 1 perceived social support from parents and friends, as well as to Time 2 depression, Time 2 subjective health complaints, and Time 2 self-harm. Gender, originally included as a predictor, did not demonstrate significance and was thus omitted from subsequent analyses. The analysis also explored two-way interactions between Time 1 perceived social support from parents and friends and Time 1 cyberbullying victimization, examining these interactions through simple slopes. Furthermore, the model was adjusted for technology use by allowing it to predict cyberbullying victimization while also controlling for face-to-face bullying victimization as a predictor of cyberbullying victimization. Additionally, Time 1 depression was controlled for in predicting Time 2 depression, and Time 1 subjective health complaints were similarly controlled for in predicting Time 2 subjective health complaints. The same procedure was followed for Time 1 self-harm and Time 2 self-harm.

## 3. Results

Correlations were computed among all this study’s variables (see Table 1). Cyberbullying victimization was negatively associated with perceived social support from parents and friends, while it was positively related to Time 1 and Time 2 depression, subjective health complaints, and self-harm. Perceived social support from parents and friends was negatively related to Time 1 and Time 2 depression, subjective health complaints, and self-harm. Time 1 depression was positively related to Time 2 depression, Time 1 and Time 2 subjective health complaints, and Time 1 and Time 2 self-harm. Time 1 subjective health complaints was correlated positively with Time 2 subjective health complaints and Time 1 and Time 2 self-harm. Time 1 self-harm was positively related to Time 2 self-harm.

### 3.1. Differences in Perceived Social Support from Parents and Friends

Two independent samples *t*-tests were performed to investigate differences in adolescents’ reports of perceived social support from friends and parents in China and in the United States. Chinese adolescents (*M* = 5.36, *SD* = 0.75) and American adolescents (*M* = 5.35, *SD* = 0.77) did not differ in their perceived social support from parents, *t*(951) = 0.18, *p* = 0.748. However, American adolescents reported more perceived social support from friends (*M* = 4.29, *SD* = 0.70) when compared to Chinese adolescents (*M* = 4.06, *SD* = 0.66), *t*(951) = 3.55, *p* = 0.001.

### 3.2. Moderation of Perceived Social Support from Parents and Friends

Confirmatory factor analysis was conducted separately for Chinese and American adolescents to validate the measurement models. Both groups demonstrated adequate model fits (for Chinese adolescents: χ^2^ = 630.26, df = 654, *p* = 0.23, CFI = 0.99, TLI = 0.99, RMSEA = 0.04, SRMR = 0.03; for American adolescents: χ^2^ = 601.94, df = 624, *p* = 0.16, CFI = 0.99, TLI = 0.99, RMSEA = 0.04, SRMR = 0.04). Standardized factor loadings were robust and statistically significant (ps < 0.001). All items served as indicators for latent variables in subsequent structural regression models.

**Figure 1:**
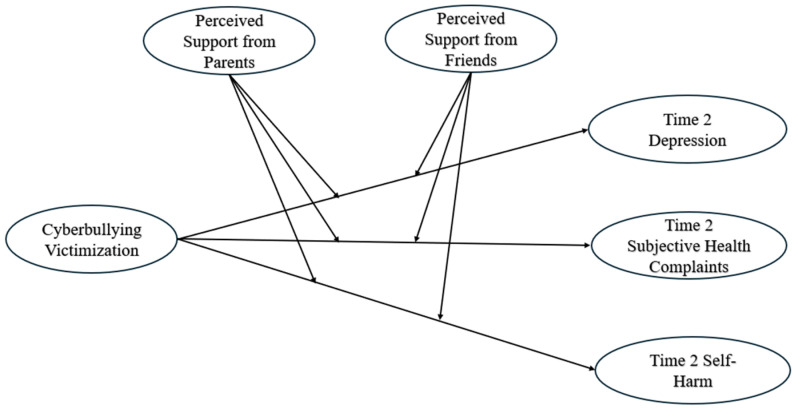
Structural regression model for the associations among Chinese and American adolescents’ cyberbullying victimization, perceived social support from parents and friends, Time 2 depression, Time 2 subjective health complaints, and Time 2 self-harm. To facilitate the reading of this figure, face-to-face bullying victimization was not included, nor was the prediction of the respective variables at Time 2 by Time 1 depression, Time 1 subjective health complaints, and Time 1 self-harm.

For Chinese adolescents (see Figure 1), as hypothesized, cyberbullying victimization showed a negative relationship with perceived social support from parents (β = −0.36, *p* < 0.001) and perceived social support from friends (β = −0.23, *p* < 0.05), controlling for face-to-face bullying victimization. Cyberbullying victimization was positively associated with Time 2 depression (β = 0.35, *p* < 0.001), Time 2 subjective health complaints (β = 0.27, *p* < 0.01), and Time 2 self-harm (β = 0.27, *p* < 0.01). Moreover, perceived social support from parents was negatively linked to Time 2 depression (β = −0.36, *p* < 0.001), Time 2 subjective health complaints (β = −0.30, *p* < 0.001), and Time 2 self-harm (β = −0.29, *p* < 0.01). Perceived social support from friends was negatively linked to Time 2 depression (β = −0.30, *p* < 0.01), Time 2 subjective health complaints (β = −0.23, *p* < 0.05), and Time 2 self-harm (β = −0.21, *p* < 0.05). Parental mediation acted as a significant moderator in the relationships between cyberbullying victimization and Time 2 depression, as well as between cyberbullying victimization and Time 2 subjective health complaints and Time 2 self-harm. Further exploration revealed that higher levels of parental mediation weakened the positive relationships between cyberbullying victimization and Time 2 depression and self-harm, whereas lower levels strengthened these relationships.

For American adolescents, cyberbullying victimization was negatively associated with perceived social support from parents (β = −0.35, *p* < 0.001) and perceived social support from friends (β = −0.33, *p* < 0.001), controlling for face-to-face bullying victimization. Cyberbullying victimization was positively correlated with Time 2 depression (β = 0.36, *p* < 0.001), Time 2 subjective health complaints (β = 0.30, *p* < 0.001), and Time 2 self-harm (β = 0.27, *p* < 0.01). Additionally, perceived social support from parents was negatively linked to Time 2 depression (β = −0.28, *p* < 0.01), Time 2 subjective health issues (β = −0.22, *p* < 0.05), and Time 2 self-harm (β = −0.21, *p* < 0.05). Perceived social support from friends was negatively linked to Time 2 depression (β = −0.36, *p* < 0.01), Time 2 subjective health complaints (β = −0.28, *p* < 0.05), and Time 2 self-harm (β = −0.25, *p* < 0.05). Similar to Chinese adolescents, parental mediation acted as a significant moderator in the relationships between cyberbullying victimization and Time 2 depression, as well as between cyberbullying victimization and Time 2 self-harm. Higher levels of parental mediation weakened the positive relationships between cyberbullying victimization and Time 2 depression and self-harm, while lower levels strengthened these relationships.

## 4. Discussion

The primary objective of this study was to investigate variations in perceived social support from parents and friends between Chinese and American adolescents and to examine whether perceived social support from parents and friends moderates the links between cyberbullying victimization, depression, subjective health complaints, and self-harm, measured one year later. This research contributes significantly to the existing literature on the role of perceived social support from parents and friends in mitigating the adverse consequences associated with cyberbullying victimization.

### 4.1. Main Effects

The purpose of this study was to explore the relationship between cyberbullying victimization, Time 2 depression, Time 2 subjective health complaints, and Time 2 self-harm for Chinese and American adolescents. Across both cultural groups, perceived social support from parents and friends exhibited a negative association with Time 2 depression, subjective health complaints, and self-harm, while cyberbullying victimization showed a positive relationship with Time 2 depression, subjective health complaints, and self-harm. These findings underscore that perceived parental support reduces vulnerability to both cyberbullying involvement and the associated negative consequences [21,29,32]. Given the active role of parents in the lives of children, adolescents, and young adults, it is plausible that parental involvement provides opportunities to discuss strategies for mitigating online risks and promoting effective coping mechanisms for managing the negative consequences associated with cyberbullying victimization. This notion is supported by research linking parental social support to decreased levels of cyberbullying involvement and negative outcomes [27,29,50,51]. Parents’ guidance and support are crucial in helping individuals navigate online environments and mitigate the negative consequences associated with cyberbullying victimization [27].

Moreover, perceived social support from friends also exhibited a negative association with cyberbullying victimization and Time 2 depression, subjective health complaints, and self-harm. These findings underscore the significant impact of supportive friendships on adolescent well-being [21,29,32,50,51]. Like parental support, friendships provide adolescents with opportunities to discuss strategies for avoiding online risks and receive support and guidance in coping with cyberbullying and its associated negative outcomes [32,50]. Supportive friendships play a pivotal role in reducing adolescents’ vulnerability to cyberbullying involvement and depression, subjective health complaints, and self-harm by fostering resilience and providing effective coping strategies. These findings highlight the critical roles that both parental support and supportive friendships play in mitigating the adverse effects of cyberbullying across different age groups, emphasizing the importance of fostering strong support networks in adolescents’ lives.

### 4.2. Buffering Effects of Perceived Social Support

Cyberbullying victimization has been consistently linked to various negative outcomes, including depression, subjective health complaints, and self-harm [8,24]. It serves as a significant stressor in the lives of adolescents, heightening their susceptibility to employing negative coping mechanisms, such as revenge-focused strategies, to alleviate the distress associated with cyberbullying [52]. Given the pervasive nature of cyberbullying and its detrimental effects, understanding factors that can mitigate these outcomes is crucial. This study focused on exploring the buffering role of perceived social support in the associations between cyberbullying involvement and depression across different age groups.

Perceived social support from parents was found to moderate the relationship between cyberbullying victimization and Time 2 depression, subjective health complaints, and self-harm for both Chinese and American adolescents. Higher levels of perceived parental support were associated with a reduction in the positive relationship between cyberbullying victimization and Time 2 depression, subjective health complaints, and self-harm, while lower levels exacerbated this relationship. This finding aligns with the literature on the buffering effects of parental support in face-to-face bullying and cyberbullying contexts [53]. Strong parental support helps adolescents feel valued and supported, enhancing their sense of efficacy in handling negative situations like cyberbullying victimization. This increased confidence likely facilitates the use of effective coping strategies [9,35]. Conversely, lower levels of perceived parental support intensify the negative consequences following cyberbullying incidents, leaving adolescents feeling more vulnerable and less capable of managing such adversities effectively.

Similarly, perceived social support from friends moderated the relationship between cyberbullying victimization and Time 2 depression, subjective health complaints, and self-harm. Higher levels of perceived social support from friends attenuated the positive association between cyberbullying victimization and Time 2 depression, subjective health complaints, and self-harm, whereas lower levels exacerbated this relationship. Friends, akin to parents, play a crucial role in adolescents’ lives by providing emotional support and practical advice on navigating challenges, including online risks [34,52]. Supportive friendships promote proactive coping strategies, thereby reducing the likelihood of negative outcomes associated with cyberbullying victimization.

Despite these similarities across both cultural groups in the buffering effects of social support, this study identified distinct impacts of parental versus friend support. Both types of support demonstrated a pattern where higher levels mitigated the adverse effects of cyberbullying victimization on Time 2 depression, subjective health complaints, and self-harm, whereas lower levels exacerbated these effects. Notably, while this pattern held true for all adolescents regarding parental and friend support, it was observed specifically among Chinese adolescents for parental support and American adolescents for friend support. Parental support plays a crucial role in both Chinese and American cultures, but the manifestation and impact of this support can vary significantly. In Chinese culture, parental involvement tends to emphasize familial harmony, filial piety, and academic achievement [41]. Parents are often seen as authoritative figures whose support is deeply rooted in maintaining family honor and ensuring children’s success, which can extend to emotional and social support in challenging situations like cyberbullying [54]. Chinese adolescents may perceive high parental support as indicative of familial cohesion and protection, enhancing their resilience against the negative impacts of cyberbullying on mental health outcomes [55]. Conversely, in American culture, parental support is often characterized by a balance between autonomy and guidance, emphasizing individualism and personal achievement [37]. American parents are encouraged to foster independence and decision-making skills in their children while providing emotional support and encouragement [56]. For American adolescents, high levels of parental support may signify a strong emotional bond and effective communication, which are essential for navigating the emotional distress caused by cyberbullying [57].

The role of peer support, particularly from friends, also differs across cultures. In Chinese culture, friendships often reflect collective values and social harmony, with an emphasis on mutual support and group cohesion [58]. Friendships among Chinese adolescents may provide emotional solace and practical advice, but the emphasis on group dynamics and conformity may influence how friends support each other in coping with cyberbullying incidents [39]. In contrast, American adolescents typically place a premium on individuality, personal choice, and peer acceptance [59]. Friendships in American culture is often viewed as platforms for self-expression, emotional sharing, and social validation, where peer support can significantly buffer the negative effects of cyberbullying by providing empathy, validation, and practical advice on managing online conflicts [60]. These cultural nuances shape how adolescents perceive and utilize parental versus friend support in coping with cyberbullying victimization. The observed pattern where high levels of parental support mitigate adverse effects across both cultures underscores the universal importance of familial bonds in providing emotional security and resilience. However, the specific impact of friend support on mitigating cyberbullying’s effects may vary culturally, reflecting differences in peer dynamics, social norms, and individualistic versus collectivist values.

### 4.3. Limitations and Future Directions

Despite this study’s contributions, its design limits insights into the longitudinal dynamics of cyberbullying, social support, and depression. Future research should adopt longitudinal approaches to discern temporal sequences and explore the roles of additional variables such as socioeconomic status and attachment dynamics with parents and peers. Furthermore, expanding assessments to include online social support would enhance understanding of how virtual interactions influence cyberbullying experiences and mitigation strategies. Additionally, future studies should broaden their focus beyond depression to encompass a wider array of negative outcomes associated with cyberbullying, thereby enriching insights into age-related vulnerabilities and protective factors.

Only the role of cyberbullying victimization was examined in this research. Follow-up research should integrate other roles, such as bystanders, to better understand how different roles in cyberbullying might relate to depression, subjective health complaints, and self-harm, and whether perceived social support might mitigate such effects. This study’s findings might not be generalized to other samples of adolescents, specifically those of different age groups and cultural values. In addition, other demographic factors, like socioeconomic status, were not assessed, although these factors could alter adolescents’ exposure to cyberbullying victimization and the availability of social support. This study relied on self-report measures of cyberbullying victimization, depression, subjective health complaints, and self-harm. Self-reports are subject to bias in reporting, and therefore, follow-up research should incorporate other forms of report, such as parent or teacher.

Future research could explore the intersection of cyberbullying victimization and adolescents’ vulnerability to fake news and racial hoaxes. Such a focus would offer additional offer valuable insights into how misinformation impacts mental health and social dynamics among adolescents.

### 4.4. Practical Implications

Educators should develop and integrate programs that foster peer support networks within schools. Encouraging collaborative activities that build strong, positive relationships among students can be beneficial. They should also provide training for teachers to recognize signs of cyberbullying and facilitate supportive discussions and interventions in the classroom. Additionally, professional development for educators on cultural differences in social support dynamics should be offered. For instance, it is important to emphasize the significance of parental support for Chinese adolescents and peer support for American adolescents. Implementing digital literacy programs that teach students about the impact of cyberbullying and strategies for seeking help is essential. These programs should include modules that highlight the importance of social support and how to access it.

Parents should be encouraged to maintain open and honest communication with their children, creating a safe space for them to share their online experiences and feelings. Educating parents on the signs of cyberbullying and the importance of providing emotional support is also important. Promoting parents’ active involvement in their children’s online lives, including understanding the platforms their children use and discussing appropriate online behavior, is necessary. Establishing support groups for parents to share experiences and strategies for dealing with cyberbullying can provide parents with a sense of community and practical advice.

Policymakers should develop policies that mandate the inclusion of anti-cyberbullying programs in school curricula. These policies should emphasize the role of social support in mitigating the effects of cyberbullying. Allocating funding for schools to implement social support programs and training for educators is important. Providing resources for the development of culturally sensitive interventions is also necessary. Launching public awareness campaigns that educate the community about the importance of social support in combating cyberbullying is crucial. These campaigns should highlight the different support needs of adolescents from diverse cultural backgrounds.

For Chinese adolescents, workshops and resources should be developed for parents to improve their understanding of cyberbullying and how they can support their children emotionally. Schools should be encouraged to organize parent–child activities that strengthen family bonds and open communication channels. Implementing school programs that engage parents in the educational process and reinforce the importance of their role in providing support against cyberbullying is essential. For American adolescents, creating peer mentoring programs where older students support younger ones can foster a culture of care and support. Encouraging extracurricular activities that build strong peer relationships and provide safe spaces for students to discuss their experiences is important. Involving community organizations in creating support networks for adolescents is crucial. This can include partnerships with local youth groups and mental health organizations. By addressing these recommendations, educators, parents, and policymakers can develop more effective, culturally sensitive interventions that leverage social support networks to mitigate the adverse effects of cyberbullying on adolescents.

## 5. Conclusions

In conclusion, this study underscores the importance of tailored interventions aimed at reducing cyberbullying involvement across diverse groups of adolescents. The findings of this study were stronger for Chinese adolescents and perceived social support from parents, while such findings were strong for American adolescents and perceived social support from friends. High levels of perceived social support from both parents and friends emerged as critical factors in mitigating the detrimental effects of cyberbullying on adolescents’ depression, subjective health complaints, and self-harm. These findings offer practical implications for developing targeted interventions that leverage social support networks to enhance resilience against cyberbullying across different cultures.

## Figures and Tables

**Table 1:** Correlation among cyberbullying bystanding.

	1	2	3	4	5	6	7	8	9	10
1. F2F BV	---									
2. CBV	0.51 ***	---								
3. SSP	−0.30 ***	−0.28 **	---							
4. SSF	−0.31 ***	−0.30 ***	0.28 **	---						
5. T1 DEP	0.31 ***	0.33 ***	−0.25 *	−0.27 **	---					
6. T2 DEP	0.30 ***	0.33 ***	−0.23 *	−0.24 *	0.60 ***	---				
7. T1 SHC	0.28 **	0.29 ***	−0.20 *	−0.23 *	0.45 ***	0.43 ***	---			
8. T2 SHC	0.28 **	0.27 **	−0.20 *	−0.24 *	0.40 ***	0.45 ***	0.58 ***	---		
9. T1 SH	0.30 ***	0.28 **	−0.21 *	−0.23 *	0.40 ***	0.41 ***	0.40 ***	0.35 ***	---	
10. T2 SH	0.27 **	0.26 **	−0.22 *	−0.20 *	0.36 ***	0.40 ***	0.42 ***	0.36 ***	0.51 ***	---
M (SD)										

Note. T1 = Time 1; T2 = Time 2; F2F BV = face-to-face bullying victimization; CBV = cyberbullying victimization; SSP = social support from parents; SSF = social support from friends; Dep = depression; SHC = subjective health complaints; SH = self-harm. * *p* < 0.05 ** *p* < 0.01 *** *p* < 0.001.

## Data Availability

Data can be requested from the first author.
